# Preliminary Evidence of EEG Connectivity Changes during Self-Objectification of Workers

**DOI:** 10.3390/s22207906

**Published:** 2022-10-17

**Authors:** Irma N. Angulo-Sherman, Annel Saavedra-Hernández, Natalia E. Urbina-Arias, Zahamara Hernández-Granados, Mario Sainz

**Affiliations:** 1 Departamento de Ingeniería Biomédica, Vicerrectoría de Ciencias de la Salud, Universidad de Monterrey, Av. Ignacio Morones Prieto 4500 Pte., San Pedro Garza García 66238, Mexico; 2 Departamento de Psicología Social y de las Organizaciones, Universidad Nacional de Estudios a Distancia, C. de Bravo Murillo 38, 28015 Madrid, Spain

**Keywords:** economical objectification, self-objectification, social neuroscience, EEG, power spectral density, partial directed coherence

## Abstract

Economic objectification is a form of dehumanization in which workers are treated as tools for enhancing productivity. It can lead to self-objectification in the workplace, which is when people perceive themselves as instruments for work. This can cause burnout, emotional drain, and a modification of self-perception that involves a loss of human attributes such as emotions and reasoning while focusing on others’ perspectives for evaluating the self. Research on workers self-objectification has mainly analyzed the consequences of this process without exploring the brain activity that underlies the individual’s experiences of self-objectification. Thus, this project explores the electroencephalographic (EEG) changes that occur in participants during an economic objectifying task that resembled a job in an online store. After the task, a self-objectification questionnaire was applied and its resulting index was used to label the participants as self-objectified or non-self-objectified. The changes over time in EEG event-related synchronization (ERS) and partial directed coherence (PDC) were calculated and compared between the self-objectification groups. The results show that the main differences between the groups in ERS and PDC occurred in the beta and gamma frequencies, but only the PDC results correlated with the self-objectification group. These results provide information for further understanding workers’ self-objectification. These EEG changes could indicate that economic self-objectification is associated with changes in vigilance, boredom, and mind-wandering.

## 1. Introduction

Economic objectification is a form of dehumanization in which workers are treated as tools for enhancing productivity [1]. In particular, tasks that divide up the labor process, such as assembly lines, can be considered objectifying activities, which are characterized as being repetitive, fragmented, and time-controlled tasks [2]. These tasks can lead to self-objectification in the workplace, which is when people perceive themselves as instruments for work [3]. The consequences of this process are severe, as it can lead to burnout, feeling emotionally drained, and a modification of self-perception in which there is a loss of human attributes, such as emotions and reasoning, while focusing on others’ perspectives for evaluating the self (for a review see [4]).

Previous studies have focused on identifying the individual’s subjective perceptions of being objectified [2,5,6] or the individual’s feeling of self-objectification [3,7,8]. These assessments rely on self-reports, which involve the use of questionnaires to acquire subjective results from the subjects of study [1,9]. However, complimenting these observations with objective physiological criteria could allow a further understanding of economic objectification. Recently, electroencephalography (EEG) was used to analyze the changes in the amplitudes of event-related potentials (ERPs) (i.e., peaks in the brain signals that are related to specific internal or external events such as stimuli or decisions) due to the presentation of sexual objectification stimuli [10,11]. However, no similar studies have been performed in the organizational context when workers perceive themselves as less than human. For this reason, there exists a need to study the underlying changes in brain activity when workers report feelings of self-objectification when performing an objectifying task. In this case, the brain activity could provide data that complement the subjective information obtained through self-reports for a better understanding of self-objectification in the workplace and the detection of different states in the individual that can be associated with the self-objectification process. An advantage of brain activity is that it does not rely on the subjective perception of the person for its measurement, and for some conditions, it can take less time or resources, or the changes in the state of the subject can be more easily detected without interrupting the task such as in real time [12,13]. However, it should be emphasized that the psychophysiological information is complementary to that obtained by subjective measures, since both provide unique, non-overlapping information [14].

The EEG was studied in terms of the changes in the neural biomarkers that provide information about either ERPs, EEG spectral coupling, or connectivity under a condition of interest [15]. ERPs are predictable waveforms that occur within temporal windows with respect to the presentation of stimuli as a consequence of neural operations. Hence, their analysis is dependent on the identification of ERP peaks over time for each stimulus [16]. In contrast, event-related synchronization (ERS) and event-related desynchronization (ERD) are not phase-locked to the event and they represent the increase or decrease in neuronal synchrony in a region, respectively, compared to a reference interval [17]. In addition, mutual synchrony between pairs of channels can be estimated using partial directed coherence (PDC), which describes the links between the activities from different brain regions and, thus, functional connectivity [18].

The present study presents an exploratory evaluation of the EEG changes that occur in participants while performing an economic objectifying task that has been previously used to create perceptions of self-objectification [19]. In particular, brain activity was analyzed over time in terms of ERS and PDC. The results were compared and correlated with the results of the self-reports of self-objectification under the hypothesis that there exist EEG features that may be useful for identifying the psychophysiological root of economic self-objectification and elucidating its effects.

## 2. Materials and Methods

### 2.1. Participants

Thirteen female undergraduate students with an age of 20.3±1.5 years (mean ± standard deviation) participated in the study. They reported no abnormal neurological conditions. The sample size was selected to obtain data for an exploratory EEG analysis of an economic objectification task. This size was comparable to other preliminary EEG studies that used PDC, as in [20]. The research protocol was approved by the Ethics Committee of the Universidad de Monterrey (protocol code 15112019-a-PSI-IBI-CEI) and was carried out in accordance with the Declaration of Helsinki.

### 2.2. Procedure

Participants sat in front of a desk with a computer and they performed an objectifying task while the EEG was recorded. To maintain attention, the experimenter stayed nearby and the room only stored the material for the experiment. Then, they answered a questionnaire for evaluating self-objectification.

#### 2.2.1. Objectifying Task and Self-Objectification Evaluation

The objectifying task was the one presented in [19]. It consisted of using a simulator of an order management job in a computer products online store. For approximately 20 min, the participants had to complete orders within the assigned time by choosing products that satisfied the requirements while adhering to the budget. In each order, the budget, requirements, and a 30 s countdown timer were displayed and the total cost of the products was calculated automatically, avoiding the need for arithmetic calculations or information memorization. If an order was fulfilled sooner, the participants waited until the timer was up for a new order to arrive. This task was repetitive, fragmented, and time-controlled so it can be considered objectifying [2]. The task efficiency, such as the time for completing each order and its correctness, was not measured.

Next, the participants answered 10 items that followed a 7-point Likert scale. Five questions asked if the user felt like a human being during the task (e.g., “a person”), whereas the remaining five asked if the participant felt like a machine (e.g., “a tool”). In order to evaluate the consistency of the answers for the human being questions and machine questions, Cronbach’s alpha was calculated for each case, resulting in a value of α=0.96 for the human being questions and α=0.94 for the machine questions. Typically, a value of α>0.7 indicates that the answers for the items of a group of questions that evaluate the same variable are related and match well so the scale of the variable that is measured is considered reliable [21].

Finally, a self-objectification index (SOI) was calculated as the arithmetic difference between the mean scores for the human being questions and machine questions [1]. Participants were labeled as `self-objectified’ subjects (SOs) if the SOI was negative and as `non-self-objectified’ (NSOs) otherwise.

#### 2.2.2. EEG Recording and Analysis

**Figure 1:**
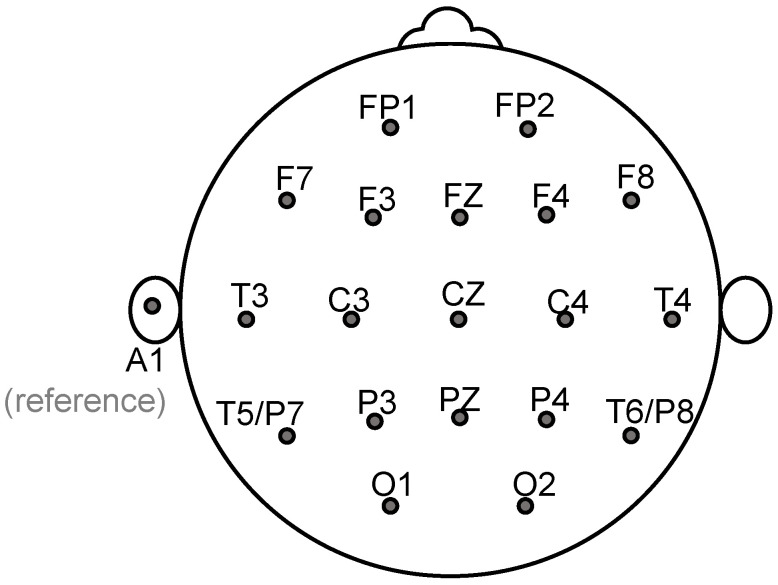
EEG electrode array.

The EEG from the 19 electrodes shown in Figure 1 was acquired using a Discovery-24 system (BrainMaster Technologies Inc., Bedford, OH, USA) and Openvibe software at 256 Hz using the left earlobe as a reference. Prior to recording, it was verified that the impedance from each sensor was below 5 kΩ to prevent poor connectivity and signal distortion [22]. MATLAB was used to perform all analyses. First, the EEG fragments greater than 100 μV and blinking and muscular artifacts were cleaned using the MWF toolbox [23], which allows artifact removal after manually selecting a sample of contaminated EEG.

Each EEG recording was divided into 3 s windows for the ERS and PDC analysis of the theta (4–7 Hz), alpha (8–13 Hz), beta (14–29 Hz), and gamma (30–80 Hz) bands. This window size was selected to ensure PDC analysis stability [18]. Analyses were limited to the first 17.5 min, which was the shortest recording length.

#### 2.2.3. ERS Analysis

The EEG was Laplacian filtered and Welch’s power spectral density (PSD) was estimated for every window. Next, the logarithmic ERS of each channel and band was calculated for each window after the first minute of recording, and the windows from the first minute were considered as the baseline to assess the ERS changes, denoted as ΔERS in the rest of the paper. This ΔERS estimation provides normal distributions and allows analysis at high frequencies [17].

Next, the existence of significant ΔERS temporal changes was evaluated in each frequency band and electrode. For this purpose, ΔERS data from each user, band, and channel were divided into 5 groups of 66 windows. In each case, data normality and homoscedasticity were evaluated for the 5-group set using Lilliefors (p<0.05) and Bartlett’s (p<0.05) tests. As the distributions were normal and heteroscedastic, Welch’s ANOVA (p<0.05) was conducted for each group set [24], followed by Games–Howell multiple comparisons (p<0.05) in case a significant difference was found [25]. If the Games–Howell comparison of the task’s end (group 5) against its beginning (group 1) was significant, the group set was labeled as an `incremental’ (synchronization enhancement or ERS) or `decremental’ trend (synchronization weakening or ERD) if the mean ΔERS in group 5 was greater or lower than that in group 1, respectively. Only these groups were used for the trend identification because the times for the beginning of the trend varied among the subjects.

Next, the electrodes and frequency bands in which at least 50% of the subjects from the same group (self-objectifying or non-self-objectifying) presented the same ERS/ERD trend were registered. This percentage threshold was proposed to consider the inter-individual differences in the EEG patterns. These electrodes and bands were considered representative of the main changes that occurred during the task so the rest of the analysis was focused on such cases.

Considering that the SOI is a continuous variable, a correlational analysis between the ΔERS data and SOI was performed. In particular, the correlation of the SOI and the arithmetic difference between the mean ΔERS of group 5 and group 1 was calculated for each representative case (band and electrode) and the significant cases (p<0.05) were registered.

### 2.3. PDC Analysis

The order of the model for the PDC estimation (p=16) was determined as the mode of the model order whose approximation provided the least mean squared error for the total number of EEG windows of all subjects [26]. Then, the PDC of each EEG window and its statistical significance were calculated for every permutation of electrode pairs on each band and subject using the AsympPDC toolbox [27]. This procedure allows for the estimation of the connectivity between all pairs of channels while considering the directionality of the interaction. The resulting 350 windows were segmented into 5 groups. Only the significant PDC values (p<0.05) were kept for each group since they were representative of brain connectivity [26].

Then, a *t*-test (p<0.05) was conducted to compare the PDC values of groups 5 and 1 for each combination of group, subject, frequency band, and electrode permutation, similar to the ERS analysis. In case a significant difference was found between both groups, it was labeled as an `incremental’ or `decremental’ connectivity trend. Additionally, the existence of significant differences between the SO and NSO groups was evaluated with a *t*-test (p<0.05). In addition, a correlational analysis (p<0.05) between the SOI and the arithmetic difference between the PDC mean values of groups 5 and 1 was performed for each electrode interaction and frequency band. Finally, a combination of the band and electrode interactions considering its directionality was labeled as representative of the objectifying task if (1) a significant temporal increment or decrement was found in at least 75% of the SOs and in a maximum of 25% of the NSOs, (2) a significant difference was observed between the SOs and NSOs, and (3) a significant correlation was found between the PDC metric and the SOI. The percentage restriction was used to consider inter-individual differences, as the EEG patterns related to the cognitive task varied among the subjects [28].

## 3. Results

Significant results for the EEG analyses were found only on the beta and gamma bands so the description of the results is focused on these bands.

### 3.1. Self-Objectification Evaluation

The self-objectification evaluation showed that eight subjects presented a negative index, whereas five subjects had a positive one. Hence, they were assigned to the SO and NSO groups, respectively. Table 1 shows the SOIs of the subjects, which are ordered according to the SOIs.

### 3.2. ERS/ERD Analysis

**Figure 2:**
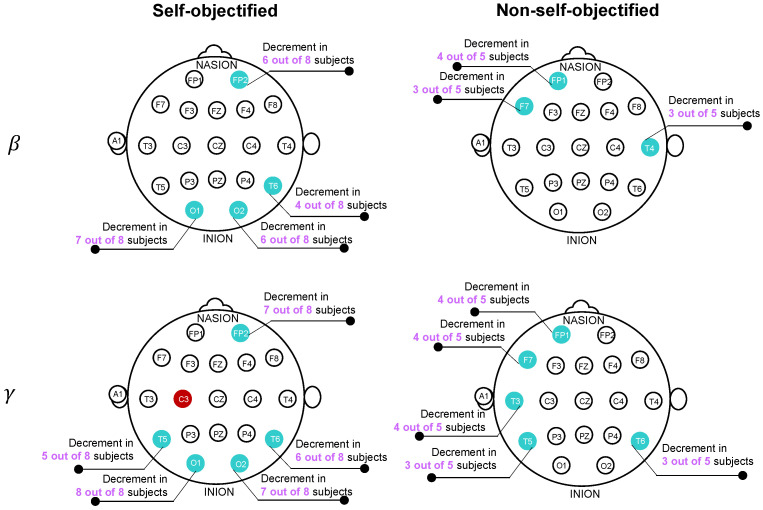
ΔERS trends in the beta band of the SOs (**top-left**) and NSOs (**top-right**) and in the gamma band of the SOs (**bottom-left**) and NSOs (**bottom-right**).

Table 2 presents the cases in which the beta or gamma ΔERS increased (ERS) or decreased (ERD) significantly throughout the task in at least half of the SOs or NSOs. For each case, the band, the sensor, the subjects and their self-objectification group, the ERS/ERD trend, and the *p*- and $\eta_{p}^{2}$ values of the comparison of groups 1 and 5 are specified (n=66 per user) and are listed in the same order as the subjects are presented. Figure 2 summarizes the number of subjects in which a significant ERS or ERD was found for the SOs and NSOs. As can be seen, the main ΔERS changes in the SO subjects involved an ERD on the bilateral temporo-occipital region on the beta and gamma bands. In the NSOs, the main changes in homoscedasticity included an ERD over the left-frontal region and the right-temporal area in both bands. Although these ΔERS differences were found for several users, they were not significantly correlated to the SOI. Note that the evaluation of the correlation of the representative cases (electrode and EEG frequency band), which were suspected to show a link between the ΔERS temporal changes and the SOI of the subjects, included a last step in which the correlation between these two variables was assessed. This last step showed no significant correlations.

Table 3 and Table 4 present the representative PDC interactions that were significantly different between the SOs and NSOs for the beta and gamma frequencies, respectively. According to the method used for selecting the representative interactions, the mean of the significant PDC values of these interactions had a significant correlation with the SOI. For each frequency band, the table shows the interactions and the *r*- and *p*-values (n=13), as well as the typical PDC tendency over time for both the SOs and NSOs. Each interaction is represented by the pair of electrodes whose functional connectivity was analyzed, along with the direction of the interaction, which is denoted by an arrow. In other terms, the EEG from the first electrode was considered to influence the one from the second electrode. In particular, the influence was expected to follow a relatively linear trend considering that the shown interactions showed a significant correlation between the SOI and the PDC metric (arithmetic difference in the connectivity of the brain activity at the end and beginning of the task). The particular values of the correlations and their significances can be seen in both tables.

**Figure 3:**
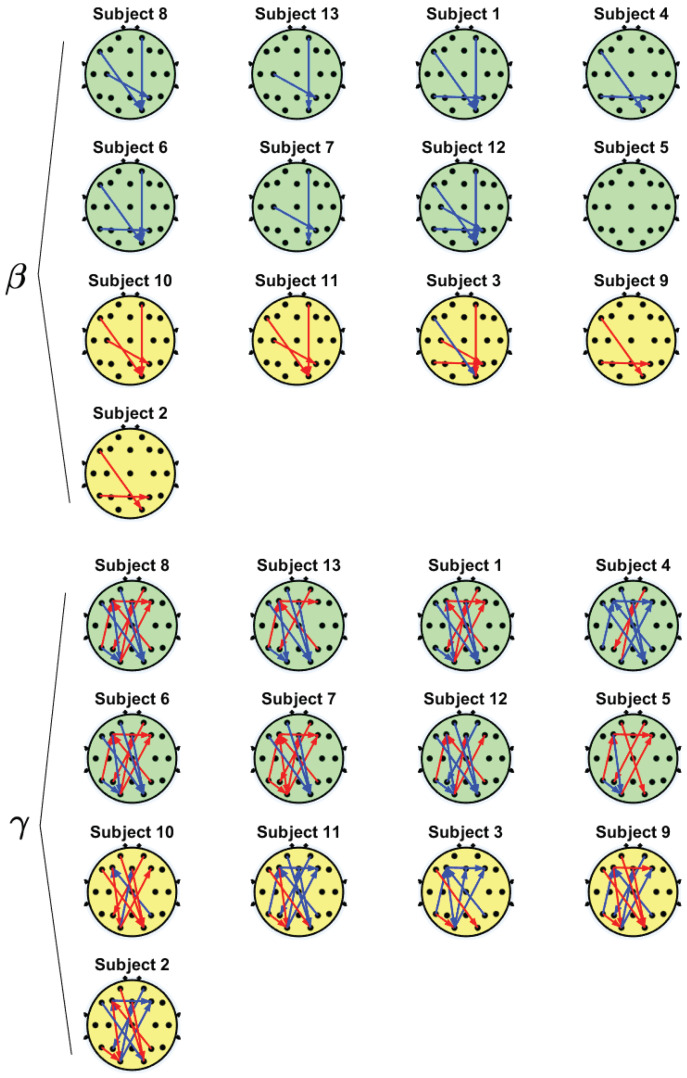
Representative PDC interactions for SOs (green) and NSOs (yellow) in the beta (**top**) and gamma (**bottom**) bands. Red and blue arrows correspond to incremental and decremental interactions (i.e, functional connectivity over time), respectively.

In addition, Figure 3 displays these representative interactions for each participant in the groups of SOs (green) and NSOs (yellow) for the beta and gamma bands. There, subjects are sorted according to their SOIs, as displayed in Table 1, and the interactions with an incremental PDC trend are represented by red arrows that indicate the direction of the functional connectivity interaction, whereas decremental interactions are shown in blue.

As can be observed in Figure 3, the beta band exhibited few electrode interactions and all of them were one-to-one (i.e., there were no networks that involved more than two electrodes). Most subjects showed fronto-occipital area interactions (F7 → O2 and Fp2 → O2). In addition, there was a representative interaction between the temporo-central and parietal areas (T5 → P4 and C3 → P4). It is important to note that these interactions are presented as PDC decrements for the SOs and as increments for the NSOs, as can be seen in Table 3.

On the other hand, the gamma frequency exhibited more interactions and there were networks in which an electrode interacted with two or more electrodes. In particular, a bidirectional interaction was observed in the fronto-occipital area (F3 → O1, O1 → Fz and O1 → F4). In addition, there was an interhemispheric interaction between the left-frontal and right-occipital areas (F7 → O2, Fp1 → O2 and Fz → O2), as well as within the frontal region (F3 → F4) and in the fronto-parietal region (P4 → F3 and Fp2 → P3). The temporal area also interacted with the frontal and occipital regions (T5 → O1 and T5 → F3). For the gamma frequency, mixed trends were observed (increments and decrements), which usually showed opposite behaviors for both groups of subjects (see also Table 4).

## 4. Discussion

The results of the self-objectification index during the economic objectifying task varied among the subjects, as can be observed in Table 1. In addition, the SOI could even be close to zero, such as in the case of Subject 5. Despite this particular subject showing a marginal self-objectification according to the procedure of the SOI estimation, the subject was kept in the subsequent analyses in order to perform the same procedure as the other subjects and to provide a more complete analysis of the correlation of the SOI and the EEG features, including these kinds of cases. It should be noted that the subsequent steps of analysis that compared the brain activities between the self-objectification groups were expected to take into consideration to some degree the inter-individual differences that could be exhibited, as in the case of this subject. Still, it is important to note that the results of the SOI indicated that performing an objectifying task did not necessarily lead to self-objectification. Even though the individuals that performed the economic objectifying task were expected to show more self-objectification compared to when a non-objectifying task is carried out, it has been discussed that factors such as mood or the awareness of being objectified may influence the results [3].

For a preliminary study of the EEG spectral and connectivity changes that occurred during the performance of the economic objectifying task, ERS and PDC analyses were conducted. For both analyses, significant results were obtained for the beta and gamma frequencies. The observations of the results in these frequencies were consistent with those of previous studies. The activity in the beta band has been associated with alertness and vigilance in some studies [29,30], whereas the gamma band has been associated with visual processing tasks [31,32].

Prior to the discussion of the ERS/ERD and PDC results, it should be mentioned that they showed no significant correlation with the SOI based on the proposed analysis. Nevertheless, they are described in the context of the PDC results, which did show a significant correlation with the SOI, to provide a more comprehensive picture of the EEG behavior in relation to the performance of the economic objectifying task. In this way, the PDC results showed a linear trend with economic self-objectification that can be used to describe the latter, although this does not imply that the ERS/ERD could not be analyzed. These results are described in the next section.

During the task, most SOs showed beta desynchronization in the parieto-occipital area (O1, O2 and T6/P8), which could be attributed to diverse cognitive processes such as working memory or decreased vigilance [29,33]. Despite parieto-occipital beta activity desynchronizing when the working memory load increased due to task complexifying [33,34], it synchronized if the task complexity remained unchanged [31], as in the present study. Conversely, parieto-occipital beta activity decreased during diminished vigilance in repetitive tasks [29], which could mean that the repetitiveness of the objectifying task may hamper vigilance in SOs due to task habituation.

Furthermore, left-frontolateral beta ERD (Fp1 and F7) in the NSOs suggests that they may have experienced mind-wandering, as this was observed during mind-wandering compared to when attention was focused on a task [32]. Similarly, a temporo-occipital gamma ERD (T5, O1, O2, and T6) in the SOs suggests that they also presented mind-wandering. Gamma power enhancement in this area is associated with the maintenance of visual information and the use of working memory [35,36]. In repetitive visual tasks, the gamma activity is attenuated when the stimulus becomes familiar [37]. Thus, these results suggest diminished attention and working memory in the SOs during the task due to mind-wandering. Mind-wandering and working memory rely on the same cognitive resources so the repeated practice of a task reduces the demand on working memory and increases mind-wandering [38,39]. Economic objectifying tasks exhibit monotonous repeatability, low complexity, high predictability, and low pace control [1]. Hence, it is reasonable that both groups of subjects experienced mind-wandering.

Additionally, both groups of subjects may have experienced boredom due to the monotony, fragmentation and repetitiveness of the task [40,41]. Boredom during the repetitive use of written material in a task is identified by the attenuation of the occipital beta and gamma bands [42], as observed in the results of the ΔERS analysis. In addition, the task of the present study is similar to vigilance tasks in its low workload [41]. Note that performing a task that is monotonous, repetitive, or has a low workload can result in the inability to sustain attention (vigilance), fatigue, and boredom or a combination of them [41]. If boredom is present, subjects can show either mind-wandering, distraction due to other tasks within the environment, or a change in the approach to the task to try to increase its difficulty [41]. The design of the experimental task prevented being distracted by another activity or interacting differently with the way the task was performed. Thus, it seems feasible that mind-wandering was a strategy used to face a task that can be considered boring, which agrees with the ΔERS results in the gamma band for the SOs and in the beta band for the NSOs. In addition, the ΔERS analysis of the beta band on the SOs suggests the possible simultaneity of vigilance deterioration and boredom, which agrees with previous studies [43,44].

In addition, the PDC analysis showed that the temporal evolution of the functional connectivity between P4 → F3 and F3 → F4 during the task was negatively correlated with the SOI. These interactions might involve components of the default mode network (DMN), in which the medial temporal and prefrontal regions interact to converge on the posterior cingulated cortex [45,46]. The DMN is associated with spontaneous cognition and becomes more active during mind-wandering and boredom [47,48]. However, the meaning of the change in these interactions is not clear since few studies have analyzed the PDC for the DMN and have focused on frequencies or experimental conditions that were different to the ones discussed [45,49,50]. In addition, both groups of subjects presented multiple bidirectional gamma interactions between the frontal and occipital areas, which intercommunicate during visual discrimination and visual search tasks [51,52], where visual stimuli are processed using involuntary exogenous attention (*bottom-up*) or volitional endogenous attention (*top-down*) [53]. Furthermore, the bidirectionality of these interactions is consistent with predictive codification theory, which proposes the simultaneous pairing of *bottom-up* and *top-down* information [54]. Note that the frontoparietal network is essential in both attentional processes and interacts with the DMN to mediate conscious states [55], though the underlying mechanisms are not fully understood [56].

Even though the ΔERS results suggest that the NSOs and SOs experienced mind-wandering, the PDC results only support this supposition for the latter. These subjects showed decremental *top-down* interactions (frontal-occipital), which could indicate task automatization by decreasing visual attention in searching the components for the order after task habituation, resulting in mind-wandering. Then, the subject could react to a task-related stimulus such as the timer, recovering the attention. This could agree with the concurrent incremental *bottom-up* interactions (occipital-frontal) that are expected during the focus on task stimuli. Note that even if the order was finished on time, the subjects had to wait until the timer stopped to continue with the next order, which could lead to mind-wandering. Still, the results suggested a greater incidence of mind-wandering over time, as reflected in the ΔERS trends.

In contrast, the NSOs presented decremental *bottom-up* interactions and inconclusive *top-down* results. This could indicate that the mind-wandering state was not interrupted in these subjects, as opposed to the SOs. Instead, these decrements could indicate the worsening of selective attention (i.e., prioritization of relevant stimuli). The gamma interactions from the visual (occipital) nodes towards the higher level (frontal) areas increased when attention was focused on visual stimuli [57,58] so the observed results may indicate that the visual information for selecting the components of the order was processed ineffectively. This agrees with the ΔERS results, which showed that these subjects presented beta and gamma desynchronization in the left fronto-lateral area, which is associated with inefficient task-related planning and reasoning [58,59].

Considering the aforementioned results, the observed EEG changes were in accordance with the those expected in tasks that lead to boredom, mind-wandering, and decreased vigilance and that share characteristics with economic objectifying tasks (i.e., repetitiveness, fragmentation, time control, and low workload).

In summary, the preliminary analysis in the present study suggests that economic self-objectification, measured through a self-objectification index, could be related to brain connectivity changes in the beta and gamma frequencies that could be associated with changes in vigilance, boredom, and mind-wandering. Then, functional brain connectivity could be studied to provide complimentary information to the measures from self-reports and, thus, could be useful for the further understanding of workers’ self-objectification.

## 5. Limitations and Future Directions

The current experimental design has some limitations, which include the lack of measurement of how well the users performed the task. This measurement is an important indicator of the cognitive processes during tasks such as working memory activation and mind-wandering. Hence, including this indicator could allow for a more extensive comparison with other studies. Another limitation is the duration of the task. Several studies that analyze vigilance and memorization are based on experiments of 20 to 30 min. Considering that the EEG recordings of the research protocol lasted approximately 17 min, it is suggested that the duration of the experiment be increased. In addition, the experimental procedure could be improved by assessing the presence of mind-wandering episodes. This could allow a better analysis of the relationship between the objective (EEG) and subjective (self-report) results and a more complete interpretation.

Furthermore, despite some expected effects, for example, boredom did not have a specific time of occurrence [41], the experiment could be extended to be longer than 20 or 30 min to improve the measure of the self-objectification effects that were reported in the present study, considering that vigilance hampering is typically reported within that timing [60]. Finally, the results of an objectifying task should be compared to those of a non-objectifying task to ensure that the results are due to the objectifying characteristics of the task (i.e., repetitiveness, fragmentation, and non-self-direction). For example, [3] analyzed two similar sales activities but varied their degree of diversity, fragmentation, and self-direction to create a non-objectifying and an objectifying task. Overall, it is necessary to conduct more studies to obtain objective evidence of the self-objectification that results from performing an economic objectifying task, as well as its effects.

In addition, a more comprehensive analysis could be achieved by measuring the task efficiency across the task (e.g., reaction time or task errors) since efficiency is a vigilance indicator that is typically correlated with the EEG modulations resulting from vigilance variations [29,42]. In contrast, efficiency is only affected by mind-wandering in demanding tasks and not simple tasks [61,62]. Hence, the real-time evaluation of the fulfilled orders would allow for the analysis of the EEG across orders and not only at the beginning and end of the task and the contextualization of the results in terms of efficiency. For this purpose, it would be recommended to assess the mind-wandering episodes to confirm the results. This could be done by interrupting the task to apply self-reports or by asking the subjects to perform a specific action when their thoughts diverge from the task [32,39].

In terms of the sample, a larger sample size that includes both male and female participants should be considered for a better analysis, considering that in the present study the volunteers were female. This is a current limitation of the results, since it cannot be assumed that the results from the study are representative of a more heterogeneous sample of people.

Finally, future studies of the relationship between brain activity and economic self-objectification should analyze the personality traits and other variables that can affect the impact of the task on the subjects, considering that the performance of the economic objectifying task had a variable impact among the subjects. This information could help to establish better thresholds or evaluate whether an individual trait could improve the predictive power of the index, as has been performed in other objectification reports [63], although they were not economic objectification studies.

## 6. Conclusions

The present paper described the effect of the psychological phenomena of self-objectification using objective measures that were obtained through the study of the brain activity (EEG) of 13 subjects while they performed an economic objectifying task. For this purpose, the changes in the ERD and PDC were analyzed by comparing them at the beginning and end of the task. Subjects were divided into two groups according to their self-objectification index, which was calculated based on a questionnaire that was given to the subjects at the end of the experiment to evaluate their self-objectification level.

The results showed that self-objectified subjects could have exhibited decreased vigilance and mind-wandering episodes due to the repetitiveness of the task. This was observed as beta and gamma ERS decrements on the temporo-occipital area, as well as incremental brain connectivity (PDC) in the gamma band between the occipital and frontal brain regions, which are associated with the bottom-up neural network. On the other hand, the non-self-objectified subjects showed ERS decrements on the frontal brain region, which could be related to planning reduction while performing the task. However, the results of these subjects were not conclusive, as mixed tendencies were observed for the top-down network. Simultaneously, the objectifying task could have generated boredom in both groups of subjects due to its characteristics (i.e., repetitiveness, fragmentation, and time control).

It should be mentioned that the present research is an exploratory study so the reported results and analysis could be used as a reference for future studies of economic self-objectification. In particular, there is a need to replicate the obtained results in order to verify that the subjects experienced the states that are indicated in the present study and to improve the experimental design based on the suggested protocol changes.

## Figures and Tables

**Table 1:** Self-objectification index (SOI) of participants.

Subject	Self-Objectification Index
8	−4.2
13	−3.4
1	−2.6
4	−2
6	−2
7	−1.6
12	−1.4
5	−0.4
10	1.6
11	3
3	3.6
9	4
2	6

**Table 2:** ERS changes that are presented in the majority of the SOs or NSOs.

Sensor	Group	Trend	Subjects (S)	*p*	$\eta_{p}^{2}$
**Beta**					
Fp1	NSOs	ERD	2, 3, 9, 10	<0.001	0.53, 0.24, 0.24, 0.33
F7	NSOs	ERD	3, 9, 10	<0.001	0.17, 0.08, 0.56
T4	NSOs	ERD	3, 10, 11	<0.001	0.25, 0.52, 0.27
Fp2	SOs	ERD	1, 4, 7, 8, 12, 13	<0.001	0.09, 0.45, 0.15, 0.29, 0.27, 0.33
O1	SOs	ERD	1, 5–8, 12, 13	S13: 0.001	0.15, 0.16, 0.64, 0.26, 0.10, 0.33, 0.09
				Other: <0.001	
O2	SOs	ERD	1, 4, 6–8, 13	<0.001	0.51, 0.34, 0.59, 0.30, 0.21, 0.12
T6	SOs	ERD	4, 6, 7, 13	<0.001	0.28, 0.17, 0.27, 0.06
**Gamma**					
Fp1	NSOs	ERD	2, 3, 9, 10	<0.001	0.53, 0.21, 0.18, 0.57
Fp2	SOs	ERD	1, 4, 5, 7, 8, 12, 13	<0.001	0.14, 0.67, 0.25, 0.16, 0.24, 0.23, 0.37
F7	NSOs	ERD	2, 3, 10, 11	<0.001	0.14, 0.29, 0.74, 0.06
T3	NSOs	ERD	3, 9, 10, 11	<0.001	0.24, 0.35, 0.82, 0.07
T5	SOs	ERD	1, 6, 7, 12, 13	<0.001	0.20, 0.45, 0.44, 0.49, 0.43
T5	NSOs	ERD	3, 9, 10	<0.001	0.38, 0.57, 0.69
T6	SOs	ERD	4, 6–8, 12, 13	<0.001	0.51, 0.44, 0.32, 0.08, 0.22, 0.13
T6	NSOs	ERD	3, 9, 10	<0.001	0.32, 0.41, 0.38
O1	SOs	ERD	1, 4–8, 12, 13	<0.001	0.61, 0.09, 0.25, 0.76, 0.32, 0.12, 0.57, 0.14
O2	SOs	ERD	1, 4, 6–8, 12, 13	<0.001	0.83, 0.63, 0.72, 0.64, 0.24, 0.46, 0.32
C3	SOs	ERS	4–8, 12, 13	<0.001	0.12, 0.08, 0.05, 0.10, 0.12, 0.17, 0.48

**Table 3:** Representative beta PDC interactions.

Interaction	*r*	*p*-Value	PDC Tendency in SOs	PDC Tendency in NSOs
F7 → O2	0.65	0.016	Decremental	Incremental
Fp2 → O2	0.66	0.014	Decremental	Incremental
T5 → P4	0.63	0.020	Decremental	Incremental
C3 → P4	0.57	0.041	Decremental	Incremental

**Table 4:** Representative gamma PDC interactions.

Interaction	*r*	*p*-Value	PDC Tendency in SOs	PDC Tendency in NSOs
F3 → O1	0.58	0.036	Decremental	Incremental
O1 → Fz	−0.76	0.003	Incremental	Decremental
O1 → F4	−0.60	0.029	Incremental	Decremental
F7 → O2	0.63	0.021	Decremental	Incremental
Fp1 → O2	0.62	0.024	Decremental	Incremental
Fz → O2	0.63	0.022	Decremental	Incremental
F3 → F4	−0.77	0.002	Incremental	Decremental
P4 → F3	−0.62	0.024	Incremental	Decremental
Fp2 → P3	−0.62	0.024	Incremental	Decremental
T5 → O1	0.62	0.025	Decremental	Incremental
T5 → F3	−0.68	0.011	Incremental	Decremental

## Data Availability

The data presented in this study are available from OSF at DOI 10.17605/OSF.IO/NCYQ3.

## Abbreviations

The following abbreviations are used in this manuscript:

EEG	Electroencepalography
ERD	Event-related desynchronization
ERS	Event-related synchronization
ERP	Event-related potential
NSOs	Non-self-objectified subjects
PSD	Power spectral density
PDC	Partial directed coherence
SOI	Self-objectification index
SOs	Self-objectified subjects
